# Association of the UCP-1 single nucleotide polymorphism A-3826G with the dampness-phlegm pattern among Korean stroke patients

**DOI:** 10.1186/1472-6882-12-180

**Published:** 2012-10-09

**Authors:** Ji Hye Lim, Mi Mi Ko, Tae-Woong Moon, Min Ho Cha, Myeong Soo Lee

**Affiliations:** 1 Medical Research Division, Korea Institute of Oriental Medicine, 1672 Yuseongdae-ro, Yuseong-gu, Daejeon, 305-811, Republic of Korea

**Keywords:** UCP-1, Polymorphism, Dampness-phlegm, A-3826G, A-1766G, Ala64Thr, Triglyceride

## Abstract

**Background:**

Patients with stroke have various syndromes and symptoms. Through pattern identification (PI), traditional Korean medicine (TKM) classifies the several syndromes and symptoms of stroke patients into five categories: Fire-heat (FH), Dampness-phlegm (DP), Yin-deficiency (YD), Qi-deficiency (QD) and Blood-stasis (BS). DP has been associated with obesity and hyperlipidemia. Uncoupling protein-1 (UCP-1), which plays a major role in thermogenesis and energy expenditure can increase the risk of obesity and can be related metabolic disorders. In this study, we elucidated the association of three polymorphisms located in the UCP-1 promoter and coding region with DP among Korean stroke patients.

**Methods:**

1,593 patients with cerebral infarction (583/DP, 1,010/non-DP) and 587 normal subjects were enrolled. The genotypes A-3826G, G-1766A and Ala64Thr (G+1068A) for each subject were determined by polymerase chain reaction with TaqMan probes and five percent of subjects were re-genotyped by sequencing method to confirm the accuracy of genotyping. The results were analyzed using a multiple logistic regression model to evaluate the genetic associations: the UCP-1polymorphisms of normal versus those of DP subjects and those of normal versus those of non-DP subjects.

**Results:**

A significantly higher percentage of subjects in the DP group possessed the A-3826G G allele than the A allele (OR=1.508, p=0.006). Furthermore, the number of subjects with the GG type of A-1766G was significantly lower in the non-DP group than the normal group in the recessive model (OR=0.606, p=0.042). In addition, an analysis of the relationship among 2 SNPs of UCP-1 and lipid serum concentration showed that the serum level of HDL cholesterol was significantly higher in subjects with the A-3826G G allele in the normal group (p=0.032). Serum triglyceride and HDL cholesterol were also associated with the A-1766G variant in the recessive model (p=0.002, p=0.046).

**Conclusions:**

These results suggest that that the A-3826G and A-1766G UCP-1 polymorphisms, which are related to obesity, might be candidate genetic markers for the DP pattern in the TKM diagnosis system.

## Background

Uncoupling protein-1 (UCP-1) is a mitochondrial transporter that is present in the mitochondrial inner membrane, and it functions in ATP synthesis by dissipating oxidative energy from brown adipose tissue (BAT) [1,2]. The metabolic activities of BAT are related to the gene quantity of UCP-1 and the extent of stimulation of the sympathetic nervous system (SNS). Thus, polymorphism of the UCP-1 gene could have an adverse effect on energy homeostasis because of reduced mRNA expression [3].

UCP-1 plays important roles in energy homeostasis, and UCP-1 gene polymorphisms have been implicated in the pathogenesis of obesity and related metabolic disorders, including lipid disorders [3,4]. An A-3826G polymorphism within the promoter region of the UCP-1 gene is a candidate gene polymorphic site related to these disorders. The G allele of A-3826G was reported to be associated with higher body fat gain, increased BMI, and reduced postprandial thermogenesis by Nagai et al. and Oppert et al. [4,5] and also known to be related with resistance against diet-induced weight loss, reduced resting energy expenditure (REE) in young women, reinforcing the metabolic action of BAT and its effect on resting energy balance in adults [6]. The A-1766G, which was found from Korean women, was also related with body fat mass and abdominal fat, and haplotype (GAG) constructed by A-3826G, A-1766G and Ala64Thr was shown to be associated with less reduction of waist-hip ratio (WHR) and body fat mass than in non-carriers among Korea women [5-7]. The Ala64Thr (G+1068A) is also known to be associated with increased WHR among Caucasians as well as lower fat content and WHR [8].

Traditional Korean medicine (TKM) is built on a medical foundation of comprehensive, integrated and holistic approaches. Pattern Identification (PI) in TKM is a unique diagnostic system that uses a comprehensive analysis on symptoms and signs [9], and is being categorized stroke into five PI: Fire-heat (FH), Qi-deficiency (QD), Dampness-phlegm (DP), Yin-deficiency (YD), and Blood-stasis (BS) [10-12]. Among these patterns, the DP pattern occurs by the prevention of Qi-blood circulation in the body, resulting in a pathological product due to various mechanisms [12]. Subjects with the DP tend to be obese and to have dyslipidemia, which is one of the causes of coronary disease [13,14].

Recently, TKM research reported a relationship between single nucleotide polymorphisms (SNP) in the Paraoxonase 1 (PON1) or Neuropeptide Y (NPY) and DP pattern [15,16]. The C-2033T and L55M polymorphisms of PON1 were related with low antioxidant capacity and serum lipid levels with obese subjects in the DP group [15]. NPY, which is widely expressed in central and peripheral nervous systems, the T allele of C-399T has a negative association with DP pattern by decreased levels of serum cholesterol, which were significantly higher in DP group [16]. In this study, we investigated the genetic distribution of the 3 SNPs of UCP-1 among Korean stroke patients with cerebral infarction (CI) DP or non-DP group and compared with normal group.

## Methods

### Study subjects

A total of 2180 Korean subjects including 587 controls without any sign of stroke and 1593 CI patients were enrolled in this study. In order to minimize the regional bias, the subjects were recruited from 10 Oriental Medical Hospitals from 2006 to 2010, including from Kyung-Hee Oriental Medical Hospital (Houeugi, Koduk in Seoul), Dae-Jeon University Oriental Medical Hospital, Wonk-Kang Oriental Medical Hospital (Jeonju, Iksan), Dong-Guk Oriental Medical Hospital (Ilsan), Kyung-Won Oriental Medical Hospital (Seoul), Dong-Sin Oriental Medical Hospital (Kwangju, Sunchon), and Sang-Ji Oriental Medical Hospital. The classification on 583 DP and 1,010 non-DP group (FH, QD, YD and BS) was performed by two TKM doctors using the Standard Pattern Identification for Stroke (K-SPI-Stroke) [17]. Patients participated in this study were diagnosed as cerebral infarction by computed tomography or magnetic resonance imaging. CI subtypes included large artery atherosclerosis (LAA), cardioembolism (CE), small vessel occlusion (SVO), stroke of other determined etiology (SOE) and stroke of undetermined etiology (SUE) subtype according to the Trial of ORG 10172 in Acute Stroke Treatment (TOAST) classification. Individuals with signs of stroke were excluded from normal groups. Subjects with infectious disease and liver diseases were also excluded. This study was approved by the Institutional Review Boards of the Korean institute of Oriental Medicine and by each of the Oriental Medical Hospitals.

### Laboratory analysis

Anthropometric parameters of each subject were obtained with a questionnaire described by Kang et al. [14]. The level of serum parameters including fasting blood sugar (FBS), total cholesterol (TC), triglyceride (TG) and high density lipoprotein (HDL-C) were determined by automated biochemical analyzer (Olympus AU400,Japan).

### Preparation of genomic DNA and identification of SNPs

Blood from each subject was collected after obtaining informed consent, and genomic DNA from each blood sample was isolated using a GeneAll genomic isolation kit (GeneAll, Seoul, Korea) according to the manufacturer’s instructions. The three SNPs of UCP-1 were genotyped using the TaqMan method with polymerase chain reaction (PCR) primers and TaqMan probes designed by ABI, Inc. (Applied Biosystems, Inc., USA). Primer information for each SNP is shown in Additional file 1: Table S1. In addition, samples for 5% of the participants were validated by sequencing to confirm the genotyping accuracy. The genotyping error rate was 0.3% according to the direct sequencing of PCR products (data not shown). The kappa value of each SNP ranged from 0.93 to 100, which indicates good accuracy. The Hardy-Weinberg equilibrium (HWE) was calculated using χ^2^ tests, and a linkage disequilibrium (LD) analysis was performed to examine the relationship between each SNP and was constructed by HapAnalyzer software [18].

### Statistical analyses

Data were statistically analyzed with SAS software, version 9.1.3 (SAS Institute, Inc., NC). Differences in continuous variables were determined by Student’s *t*-test or Wilcoxon rank-sum test after confirming normality by a Kolmogorov-Smimov test. Categorical variables were compared with a Chi-squared test or Fisher’s exact test. Estimation of the association of SNPs with DP was performed by multiple logistic regression, which was adjusted for sex, age, smoking status and drinking status, as well as odds ratios (ORs), with 95% confidence intervals (95% CI). The effect of the A-3826G and A1766G polymorphisms on serum lipid and sugar in the normal group was evaluated by a general linear model after adjusting for sex, age, smoking status and drinking status. Statistical significance was set at p < 0.05.

## Results

**Figure 1:**
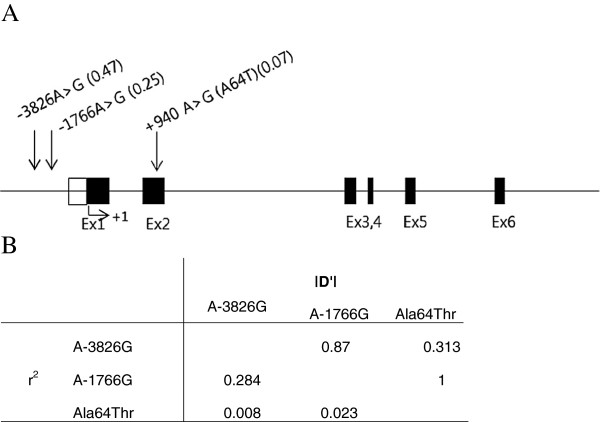
**Polymorphisms in UCP-1.A**) A map of the SNPs located in the 4q28-q31 locus of the UCP-1 gene. Minor allele frequencies are shown in parentheses. The translation start site is indicated by +1. Open and closed boxes represent the untranslated and translated regions, respectively. **B**) Linkage distribution among the three SNPs.

The demographic and clinical characteristics of CI patients classified as DP group or Non-DP group and normal subjects are summarized in Table 1. The results showed that body characteristics (weight, waist, and WHR) values in DP group were significantly higher than those in normal group (p< 0.0001). Additionally, serum lipid parameter (total cholesterol and triglyceride) levels were higher in the DP group than those in non-DP group. General characteristics of subjects in each pattern of Non-DP group are shown in Additional file 2: Table S2. The locations of 3 SNPs within the UCP-1 gene are shown in Figure 1(A), and their characteristics are listed in Table 2. Two of these SNPs are located in the UCP-1 promoter region, and the other SNP is located in exon 2. All of the studied SNPs satisfied the Hardy-Weinberg equilibrium (p > 0.05) according to the International HapMap Project [17]. The LD coefficients between the 3 SNPs are shown in Figure 1(B). The value of *r*^*2*^ was 0.008-0.284, which means that the three SNPs were not linked. These results are consistent with previous reports, and the SNP distribution in normal subjects was polymorphic and in accord with the HWE in Table 2. Table 3 shows the allele and genotype distribution of the three SNPs in the non-DP and DP groups compared with the normal groups. The percentage of subjects with the G allele of A-3826G was significantly higher in the DP group than in the normal group in the dominant model (77.76% in dampness-phlegm vs. 71.77% in normal, OR = 1.508, p = 0.006, power=85.3%). In other hand, G allele of A-1766G and A allele of Ala64Thr were previously revealed to be highly related to obesity showed little relation with obesity in this study of comparing the DP group with normal group. However, the G allele frequency of A-1766G was significantly lower in the non-DP group than the normal group in the recessive model (4.77% in non-dampness-phlegm vs. 5.10% in normal, OR = 0.606, p =0.0423, power=56.9%).

**Table 1:** General characteristics of the study subjects

**Characteristic**	**Normal (N)**	**Non-dampness-phlegm (N)**	***Sig.***^***a***^	**Dampness-phlegm (N)**	***Sig.***^***b***^
Sex (M/F)	273/314	619/523	0.0024	314/331	0.4453
Age (year)	64 (58, 69)	70 (60, 76)	<0.0001	69 (61, 75)	<0.0001
TOAST (LAA/CE/SVO/SOE/SUE)	-	248/77/617/17/48		128/31/386/26	
Weight (kg)	62.0 (56.0, 69.0)	60.0 (53.0, 68.0)	0.0001	63.0 (56.0, 70.0)	0.0461
BMI (kg/m^2^)	24.17 (22.65, 26.02)	23.28 (21.29, 25.39)	<0.0001	24.6 (22.5, 26.7)	0.0588
Waist (cm)	85.0 (79.0, 90.0)	86.0 (80.0, 92.0)	0.0032	89.0(82.0, 95.0)	<0.0001
Hip (cm)	96.0 (92.0, 100.0)	93.0 (87.5, 98.0)	<0.0001	95.0 (89.0, 99.0)	0.0002
WHR	0.88 (0.85, 0.91)	0.93 (0.89, 0.97)	<0.0001	0.94 (0.90, 0.97)	<0.0001
Smoking (Y/N)	56/531	292/850	<0.0001	153/491	<0.0001
Drinking (Y/N)	195/392	416/726	<0.0001	196/447	0.0206
GOT (U/ml)	23.0 (18.0, 29.0)	22.1 (18.0, 29.0)	0.0001	23.0 (18.0, 28.1)	0.0005
GPT (U/ml)	21.0 (16.0, 30.0)	19.0 (14.0, 29.0)	<0.0001	19.0 (14.0, 28.0)	<0.0001
T-Cholesterol (mg/dL)	199.0 (175.0, 226.0)	180.0 (152.0, 210.0)	<0.0001	189.0(160.0, 219.0)	<0.0001
Trig (mg/dL)	131.0 (91.0, 182.0)	127.0 (90.0, 185.0)	0.8524	132.0 (94.0, 195.0)	0.1055
HDL (mg/dL)	51.0 (43.2, 59.9)	42.0 (35.0, 50.0)	<0.0001	41.0 (33.5, 48.8)	<0.0001
FBS (mg/dL)	99.0 (92.0, 106.0)	105.0 (93.0, 132.0)	<0.0001	107.0 (94.0, 128.0)	<0.0001
BUN	14.5 (12.0, 17.6)	14.0 (11.5, 18.0)	0.3859	13.9 (11.0, 17.1)	0.0031
Cr	0.90 (0.70, 1.00)	0.90 (0.70, 1.00)	0.521	0.80 (0.70, 1.00)	0.0002

* Data were expressed as frequencies for categorical variables and as the mean*±* standard deviation for continuous variables.WHR: waist hip ratio. TOAST: trial of ORG 10172 in acute stroke treatment. LAA: large-artery atherosclerosis. CE: cardio embolism. SVO: small vessel occlusion. SOE: stroke of other etiology. SUE stroke of undetermined etiology. GOP: glutamate oxaloacetate transaminase. GPT: glutamate pyruvate transaminase. Trig: Triglyceride sig*.*a: *P* value of normal versus non-dampness-phlegm using a Chi-squared test for categorical variables and the Wilcoxon two-sample test for continuous variables. sig*.*b: *P* value of normal versus dampness-phlegm using a Chi-squared test for categorical variables and the Wilcoxon two-sample test for continuous variables.

**Table 2:** Characteristics of SNPs identified by genomic sequencing of the promoter and exon of UCP-1 in the normal group

**SNP**	**Rs No.**	**Position**	**Nucleotide change**	**Genotype distribution (N)**			**Minor allele frequency**	**HWE p**
				**CC**	**CR**	**RR**		
A-3826G	rs1800592	Promoter	A>G	126	291	164	0.4673	0.8845
A-1766G	rs3811791	Promoter	A>G	335	201	43	0.2478	0.0974
Ala64Thr	rs45539933	Exon2	A>G	508	77	0	0.0658	0.0883

**Table 3:** The genotype distribution of UCP-1 polymorphisms in the dampness-phlegm and non-dampness-phlegm groups compared with the normal group

**Model**	**SNPs**	**Genotype**	**Normal**	**Non-dampness**	**OR [95% CI]**	***p***	**Dampness**	**OR [95% CI]**	***p***
				**phlegm**			**Phlegm**		
Allele	A-3826G	A	619 (53.27)	1,032 (51.43)	1.097	0.2586	575 (49.57)	1.205	**0.0443**
		G	543 (46.73)	978 (48.66)	(0.934,1.290)		585 (50.43)	(1.005,1.444)	
	A-1766G	A	871 (75.22)	1512 (75.6)	0.968	0.7371	873 (75.91)	0.98	0.8548
		G	287 (24.78)	488 (24.4)	(0.802,1.169)		277 (24.09)	(0.793, 1.212)	
	Ala64Thr	A	77 (6.58)	130 (6.45)	0.984	0.9226	91 (7.84)	0.879	0.468
		G	1,093 (93.42)	1,886 (93.55)	(0.710,1.364)		1,069 (91.67)	(0.621,1.245)	
^†^Do	A-3826G	AA	164 (28.23)	267 (26.57)	1.168	0.2319	129 (22.24)	1.508	**0.0065**
		AG+GG	417 (71.77)	738 (73.43)	(0.906,1.506)		451 (77.76)	(1.122, 2.028)	
	A-1766G	AA	335 (57.86)	563 (56.3)	1.066	0.5872	328 (57.04)	1.023	0.8644
		AG+GG	244 (42.14)	437 (43.7)	(0.846,1.344)		247 (42.96)	(0.789, 1.327)	
	Ala64Thr	GG	508 (86.84)	879 (87.2)	0.989	0.9489	492 (84.83)	1.087	0.6157
		GA+AA	77 (13.16)	129 (12.8)	(0.704,1.389)		88 (15.17)	(0.762, 1.583)	
^†^R	A-3826G	AA+AG	455 (78.31)	765 (76.12)	1.093	0.5257	446 (76.90)	1.092	0.5749
		GG	126 (21.69)	240 (23.38)	(0.831,1.437)		134 (23.10)	(0.803,1.485)	
	A-1766G	AA+AG	536 (92.57)	949 (94.9)	0.606	**0.0423**	545 (94.78)	0.801	0.4183
		GG	43 (7.43)	51 (5.1)	(0.374, 0.983)		30 (5.22)	(0.468,1.371)	
	Ala64Thr	GG+GA	585 (100)	1,007 (99.9)	ND	ND	577 (99.48)	ND	ND
		AA	0 (0)	1 (0.1)			3 (0.52)		

* The values indicate the frequency of subjects (%). OR (95% CI) and Siga, Sigb value were calculated using a binary logistical regression model adjusted for sex, age, smoking, and drinking with normal versus non-dampness-phlegm, normal versus dampness-phlegm . *†*Do and R denote dominant and recessive models, respectively. CI: confidence interval. *P*-values with statistical significance were presented in bold (*<*0*.*05).

To confirm which factor was affected by A-3826G or A-1766G, we compared serum lipid parameters according to genotype in normal subjects (Table 4). A-3826G was associated with serum HDL-C. The mean serum HDL-C of the subjects with the GA and GG type at the −3826 position of the UCP-1 gene was 52.74 mg/dL and 54.63 mg/dL, respectively. These means were significantly higher than the mean serum HDL-C (50.92 mg/dL) of the subjects with the AA type in the dominant model (p = 0.0317, power=50.3%). The level of serum triglycerides (p = 0.0229, power=97.5%) and HDL-C (p = 0.0465, power=39.3%) was associated in the recessive model. However, parameters on obesity and serum lipids were not significantly different between DP group and non-DP group (data not shown).

**Table 4:** Association analysis of serum biochemical parameters by genotype of UCP-1 among the normal group

**Parameter**	**Genotype**						***P***			
	**A-3826G**			**A-1766G**			**A-3826G**		**A-1766G**	
	**AA**	**AG**	**GG**	**AA**	**AG**	**GG**	**Do**	**R**	**Do**	**R**
n	164	291	125	335	200	43				
BMI (kg/m2)	24.44±2.76	24.30±2.56	24.59±3.01	24.30±2.68	24.49±2.81	24.70±2.66	0.9351	0.4065	0.3249	0.463
TC (mg/dl)	201.52±43.28	204.15±37.74	196.79±37.72	202.46±40.18	201.52±38.82	196.98±38.69	0.8485	0.0708	0.5684	0.3833
TG (mg/dL)	151.66±89.69	149.56±78.51	137.61±78.32	150.28±74.70	147.42±84.47	111.60±58.36	0.4427	0.1699	0.2031	**0.0028**
HDL-cholesterol (mg/dl)	50.92±12.41	52.74±12.79	54.63±14.26	51.63±12.97	53.37±12.89	56.43±14.36	**0.032**	0.0847	0.0379	**0.0465**

The value indicates the number of subjects (mean±SD). The p value was calculated using a general linear model adjusted for sex, age, smoking, and drinking. Statistical significance was presented in bold (<0.05). Do, and R denote dominant, and recessive models, respectively.

## Discussion

A recent study published by Kim et al. explained that dampness-phlegm is an impediment to Qi, energy that circulates in channels called meridians and that causes various symptoms and signs when blocked or disrupted because of its high turbidity, heaviness, stickiness, and downward flowing properties [12,19]. In addition, DP was related to clinical indicators, such as overweight, pale tongue, and slippery pulse [12]. In TKM, DP has long been a suspected as a cause for obesity. Dong-eui-bo-gam, which are the most extensively read medical texts by TKM doctors and are praised as one of the most important medical texts in TKM [20], declared dampness-phlegm as a main factor of obesity, stating that overweight people have an abnormal biomechanical flow and that the Qi deficiency generates cold, cold generates dampness, and dampness generates phlegm, which finally leads to obesity [21]. Recently, there have been several scientific attempts to verify the relationship between DP and obesity. The study of the relationship of DP tongue diagnosis to hyperlipidemia in stroke suggests that serum lipid levels, such as total cholesterol, HDL cholesterol, and triglyceride, were higher in the DP group than those in the non-DP group [12]. In addition, Min et al. reported that TC, BMI and waist circumference were significantly increased in DP patients [22]. A case–control study of the relationship of DP and blood lipid levels revealed that DP was significantly correlated with increased LDL cholesterol and was an independent predictor of hyperlipoproteinemia [23]. However, few studies using a genetic analysis to prove the relationship between DP and obesity have been carried out up to now. An analysis on obesity-related genotype can provide a significant insight to present scientific evidence including a number of clinical trials that show the relationship between DP and obesity. UCP-1 is a major obesity-related gene that regulates energy homeostasis inside body [24]. When exposed to coldness, the expression of UCP-1 is promoted by adaptive thermogenesis, that is, heat generation consuming pH-gradient induced by oxidative phospholyation and by increased adrenergic stimulation, β3-agonists, retinoids, thyroid hormone, PPARγ ligand and leptin [25,26].

However, the inhibition of gene expression or gene activity caused by genetic deficiency of UCP-1 may result in imbalance on heat generation inside body, which changes in the oxidation of free fatty acids in the mitochondria can alter the blood levels of lipids through tissue cholesterol transport and ultimately lead to obesity. Therefore, this mechanism to control blood lipids may be affected by genetic polymorphisms such as A-3826G polymorphism of UCP-1 gene [27]. This was proved by a study that showed lowered activation of sympathetic nerve and declined expression of UCP-1 in BAT of obese rats and by another in vivo study that reported overexpression of UCP-1 reduced degree of obesity induced by high fat diet. This signifies the acceleration of energy consumption by the expression of UCP-1 is effect**i**ve to the prevention and suppression of obesity [28,29].

The A-3826G, A-1766G and Ala64Thr, polymorphisms of UCP-1 gene, were previously associated with weight loss, abdominal obesity and metabolic disorder, which are risk factors of stroke. In the current study, we examined that A-3826G SNP were significantly associated with DP among Korean stroke patients. The G allele frequency of A-3826G was significantly higher than the A allele in the DP group (p=0.0247), and the number of subjects with the GG or GA genotype was larger than those with the AA type. A large distribution of G allele revealed after the analysis on A-3826G polymorphism among the patients with DP is also found among the obese [30]. Considering DP acts as a causative factor of obesity in TKM, and UCP-1 is regulator of obesity, DP and UCP-1 seem to be highly associated each other. Investigating the genetic polymorphic distribution is meaningful to predict and diagnose obesity.

Oberkoflr et al. reported that UCP-1 mRNA levels in visceral fat were lower in obese subjects and that UCP-1 mRNA levels in fat tissue were regulated by the UCP-1 genotype [31,32]. This result may have been observed because the G allele carrier of A-3826G in UCP-1 regulated by the extent of obesity may have reduced expression, which causes less energy dissipation as heat.

This study has several limitations. The first limitation relates to the diagnostic standard for PI. The PI types were confirmed by two experts with sufficient clinical experiences. Nevertheless, their diagnoses did not match. PI still has limitations in consistency and reproducibility because of its high dependence on subjective diagnostic indicators. Second, although DP in TKM is generally considered as a major causative factor for obesity, DP is not the sole cause that induces obesity, and other pattern such as Fire-Heat has also potential to result in obesity [33]. Third, sample size was not enough to generalize the association between UCP-1 polymorphisms and DP. Especially, the number of subjects in normal group was smaller than that of the patients group. Thus, a further study to confirm the association will be needed.

We already reported that polymorphisms of the PON1 and NPY genes, which were associated with stroke and obesity, were related to DP among Korean stroke patients. The results of this study replicate the relationship that other studies have found between genetic polymorphism and PI, and the A-3826G SNP of the UCP-1 gene might be a predictive genetic marker for DP pattern.

## Conclusions

We showed an association of UCP1 polymorphisms with DP in Korean stroke patients. A-3826G and A-1766G UCP-1 polymorphisms, which are related to obesity, might be candidate genetic markers for DP pattern in the TKM diagnosis system.

## Competing interests

The authors declare that they have no competing interests.

## Authors’ contributions

JHL: performance of experiments, data analysis, writing manuscript. MMK: data analysis (statistics). TWM and MSL: design and coordination and helped to draft the manuscript. MHC: experimental design, performance of experiments, writing manuscript. All of the authors read and approved the final manuscript.

## Pre-publication history

The pre-publication history for this paper can be accessed here:

http://www.biomedcentral.com/1472-6882/12/180/prepub

## Supplementary Material

Additional file 1 **Table S1. **Primer sets for PCR amplification and genotyping of each SNP. Click here for file

Additional file 2 **Table S2.** General characteristics of Non-dampness-phlegm pattern subjects. Click here for file
