# Supplementary material for: Association of the UCP-1 single nucleotide polymorphism A-3826G with the dampness-phlegm pattern among Korean stroke patients
Source: BMC Complement Altern Med. 2012 Oct 9;12:180. doi: 10.1186/1472-6882-12-180 (PMC3537753; doi:10.1186/1472-6882-12-180)
Supplement: Additional file 1 — Table S1. Primer sets for PCR amplification and genotyping of each SNP. [file 1472-6882-12-180-S1.doc]

Supplement table 1. Primer sets for PCR amplification and genotyping of each SNP.

| SNP | Assay Name | Forward Primer Name | Reverse Primer Name | Probe 1 Name | Probe 1 Dye | Probe 2 Name | Probe 2 Dye |
| --- | --- | --- | --- | --- | --- | --- | --- |
| A-3826G | rs1800592 | C8866368_20_F | C8866368_20_R | C8866368_20_V | VIC | C8866368_20_M | FAM |
| A-1766G | rs3811791 | C2052379_10_F | C2052379_10_R | C2052379_10_V | VIC | C2052379_10_M | FAM |
| Ala64Thr | rs45539933 | C25619416_30_F | C25619416_30_R | C25619416_30_V | VIC | C25619416_30_M | FAM |
