# Supplementary material for: Association of the UCP-1 single nucleotide polymorphism A-3826G with the dampness-phlegm pattern among Korean stroke patients
Source: BMC Complement Altern Med. 2012 Oct 9;12:180. doi: 10.1186/1472-6882-12-180 (PMC3537753; doi:10.1186/1472-6882-12-180)
Supplement: Additional file 2 — Table S2. General characteristics of Non-dampness-phlegm pattern subjects. [file 1472-6882-12-180-S2.doc]

Supplement table 2. General characteristics of Non-dampness-phlegm pattern subjects

| Characteristic | QD | YD | FH |
| --- | --- | --- | --- |
|
| Sex (M/F) | 121/235 | 84/108 | 346/115 |
| Age (year) | 70 (63, 78) | 74 (66, 79) | 69 (60, 75) |
| TOAST | 67/24/237/6/18 | 49/14/113/5/11 | 131/39/265/6/18 |
| (LAA/CE/SVO/SOE/SUE) |
| Weight (kg) | 57.0 (50.0, 63.0) | 56.3 (50.0, 64.0) | 65.0 (57.0, 72.0) |
| BMI (kg/m^2^) | 22.66 (20.83, 24.46) | 22.51 (20.52, 24.69) | 24.10 (22.09, 26.11) |
| Waist (cm) | 85.5 (80.0, 91.0) | 83.0 (76.0, 90.0) | 88.9 (82.0, 94.0) |
| Hip (cm) | 93.0 (87.0, 97.0) | 91.0 (85.0, 97.0) | 94.0 (90.0, 98.0) |
| WHR | 0.89 (0.86, 0.92) | 0.93 (0.89, 0.96) | 0.94 (0.90, 0.98) |
| Smoking (Y/N) | 61/295 | 29/163 | 168/293 |
| Drinking (Y/N) | 83/323 | 48/144 | 218/243 |
| GOT (U/ml) | 22.0 (18.0, 28.0) | 21.0 (18.0, 28.0) | 23.0 (19.0, 31.0) |
| GPT (U/ml) | 18.0 (13.0, 27.0) | 17.0 (13.0, 25.0) | 21.0 (15.0, 31.0) |
| T-Chol (mg/dL) | 180.5 (155.0, 212.0) | 185.0 (162.5, 212.5) | 178.0 (147.0, 208.5) |
| Trig (mg/dL) | 120.0 (88.0, 176.0) | 124.0 (85.0, 174.0) | 129.5 (92.0, 192.0) |
| HDL (mg/dL) | 42.9 (36.0, 53.0) | 43.0 (37.0, 49.9) | 41.0 (34.0, 48.0) |
| FBS (mg/dL) | 102.0 (91.0, 130.0) | 106.0 (98.0, 141.0) | 105.0 (94.0, 126.0) |
| BUN | 14.0 (12.0, 18.5) | 14.4 (11.3, 18.6) | 14.0 (11.5, 18.0) |
| Cr | 0.80 (0.70, 1.00) | 0.80 (0.70, 1.00) | 0.90 (0.78, 1.10) |

All values are of the Median (Q1, Q3) otherwise indicated; Q1: 25th percentile, Q3: 75th percentile;
